# DCANet: Disentanglement and Category-Aware Aggregation for Medical Image Segmentation

**DOI:** 10.3390/s26041300

**Published:** 2026-02-17

**Authors:** Xiaoqing Li, Hua Huo, Chen Zhang

**Affiliations:** Information Engineering College, Henan University of Science and Technology, Luoyang 471000, China

**Keywords:** transformer, medical image segmentation, deep learning, boundary ambiguity

## Abstract

Medical image segmentation is essential for clinical decision-making, treatment planning, and disease monitoring. However, ambiguous boundaries and complex anatomical structures continue to pose challenges for accurate segmentation. To address these issues, we propose DCANet (Disentangled and Category-Aware Network), a novel framework that effectively integrates local and global feature representations while enhancing category-aware feature interactions. In DCANet, features from convolutional and Transformer layers are fused using the Feature Coupling Unit (FCU), which aligns and combines local and global information across multiple semantic levels. The Decoupled Feature Module (DFM) then separates high-level representations into multi-class foreground and background features, improving discriminability and mitigating boundary ambiguity. Finally, the Category-Aware Integration Aggregator (CAIA) guides multi-level feature fusion, emphasizes critical regions, and refines segmentation boundaries. Extensive experiments on four public datasets—Synapse, ACDC, GlaS, and MoNuSeg—demonstrate the superior performance of DCANet, achieving Dice scores of 84.80%, 94.07%, 94.60%, and 79.85%, respectively. These results confirm the effectiveness and generalizability of DCANet in accurately segmenting complex anatomical structures and resolving boundary ambiguities across diverse medical image segmentation tasks.

## 1. Introduction

Medical image segmentation, as a critical component of computer-aided diagnosis, plays a vital role in clinical decision-making, treatment planning, and disease monitoring. In recent years, deep learning-based approaches have achieved remarkable progress in this field, with convolutional neural networks (CNNs) and Transformer-based architectures significantly improving segmentation accuracy. However, in multi-organ and multi-tissue segmentation tasks, several challenges remain unresolved. This ambiguity not only leads to inaccurate predictions but also greatly limits the robustness and clinical reliability of existing segmentation models in complex medical imaging scenarios. The rapid advancement of deep learning has shown tremendous potential in addressing this challenge. Among existing approaches, the U-Net architecture, with its encoder–decoder structure and skip connections, has achieved remarkable success by enabling effective multi-scale feature fusion. Building upon this foundation, numerous variants have been proposed, including UNet++ [1], Attention U-Net [2], and Res-UNet [3], as well as task-specific networks such as PraNet [4] and CASF-Net [5], all of which have demonstrated strong performance in organ segmentation and lesion detection. Although recent methods such as HyperSegNAS [6] and ADWU [7] have achieved impressive results, they tend to overlook boundary-specific information that is essential for accurate organ segmentation. Despite these advances, medical image segmentation still faces a persistent challenge—boundary ambiguity. Such ambiguity, especially prevalent between organs with similar intensity distributions, makes it difficult for models to precisely delineate anatomical structures, thereby limiting the reliability and robustness of segmentation outcomes in complex clinical scenarios.

To mitigate boundary ambiguity, several studies have focused on boundary-aware optimization strategies. For example, [8] optimizes the Hausdorff distance to enhance boundary accuracy, while [9] introduces a boundary-based loss to improve segmentation performance around object contours. Although these methods have achieved certain improvements, they also suffer from notable drawbacks. Specifically, the design of boundary-oriented loss functions often increases computational complexity, making model training more time-consuming and optimization more unstable. Consequently, their scalability and applicability to large-scale medical segmentation tasks remain limited.

To tackle the aforementioned challenges, we propose a novel medical image segmentation framework named DCANet (Feature Coupling and Category-Aware Aggregation Network). The core idea of DCANet is to enhance feature representation and alleviate boundary ambiguity by disentangling category-specific features and performing adaptive multi-level aggregation under category guidance. Specifically, a Feature Coupling Unit (FCU) is designed to effectively integrate convolutional and transformer representations, achieving complementary global and local feature modeling. On this basis, we propose a Decoupled Feature Module (DFM) to explicitly separate encoder features into foreground and background representations, thereby enhancing the discriminability of organ boundaries and reducing inter-class confusion. Furthermore, a Category-Aware Integration Aggregator (CAIA) is introduced to adaptively fuse multi-level features under the guidance of category-aware cues. By incorporating category-specific and context-aware information, CAIA refines semantic alignment and strengthens inter-class separability during feature fusion.

The main contributions of this work are summarized as follows:

- We design a Feature Coupling Unit (FCU) that couples convolutional and transformer features, enabling complementary global–local representation learning.
- We develop a Decoupled Feature Module (DFM) that explicitly disentangles deep encoder features into multi-category foreground and background components, improving boundary discriminability.
- We introduce a Category-Aware Integration Aggregator (CAIA) that adaptively fuses multi-level features guided by category-aware cues, enhancing inter-class separability and semantic consistency.
- Extensive experiments on four public datasets (Synapse, ACDC, GlaS, and MoNuSeg) demonstrate that DCANet achieves superior performance and generalization capability across diverse medical image segmentation tasks.

The remainder of the paper is organized as follows. Related works are discussed in Section 2. Section 3 introduces the proposed method, while Section 4 presents the experimental setup, results, and qualitative analysis of the experimental findings, including ablation experiments. Section 5 concludes the paper.

## 2. Related Work

CNN-based Medical Image Segmentation:Medical image segmentation, which aims to accurately separate organs or lesions from the background, is a fundamental task in medical image analysis. Traditional handcrafted methods struggle with challenges such as noise, uneven contrast, and blurred boundaries. In recent years, convolutional neural networks (CNNs) have significantly advanced this field, with U-Net [10] serving as a milestone model due to its encoder–decoder architecture and skip connections. Numerous variants, including UNet++ [1], Attention U-Net [2], Res-UNet [3], and DenseUNet [11], have further improved segmentation performance in multi-organ and lesion segmentation tasks. However, CNNs remain constrained by their local receptive fields, limiting their ability to capture long-range dependencies and global context. Moreover, inherent challenges in medical images, such as boundary ambiguity and organ variability, still lead to unstable segmentation results.

Transformer-based Medical Image Segmentation: Transformers, originally designed for natural language processing, have recently gained traction in computer vision. The Vision Transformer (ViT) [12] pioneered this direction by dividing images into patches and feeding them as sequences, enabling effective long-range dependency modeling and achieving promising results in classification and segmentation. Building on ViT, several variants have been introduced to better suit medical image segmentation. For instance, Swin Transformer [13] employs hierarchical window attention to balance accuracy and efficiency, and has been successfully applied to organ and lesion segmentation. Pyramid Vision Transformer (PVT) [14] introduces a pyramid structure for multi-scale feature extraction, making it suitable for high-resolution and structurally complex medical images. SegFormer [15] combines a lightweight design with strong representation capability, achieving competitive performance across diverse segmentation benchmarks. These Transformer-based methods provide advantages over CNNs in capturing global context and multi-scale representations, offering new opportunities for medical image segmentation. However, their high computational cost and reliance on large annotated datasets limit their applicability in clinical scenarios with limited data. Thus, developing models that preserve global modeling ability while improving efficiency and generalization remains an open challenge.

Addressing Boundary Ambiguity: Boundary ambiguity remains a critical challenge in medical image segmentation. To address this issue, researchers have proposed various strategies. For example, some studies [16] leverage cross-level feature aggregation to integrate low-level structural details with high-level semantic representations, thereby enhancing boundary delineation. Others [17] introduce multi-scale edge-guided attention mechanisms to improve the model’s capability in capturing weak or ambiguous boundary regions. Although these approaches have achieved notable progress in alleviating boundary ambiguity, they often rely on complex aggregation or attention designs, which increase computational overhead. Moreover, their generalization ability across different imaging modalities and complex clinical scenarios remains limited. Therefore, developing efficient and robust models to more effectively address boundary ambiguity continues to be a significant research focus in medical image segmentation.

## 3. Method

### 3.1. Network Architecture

**Figure 1:**
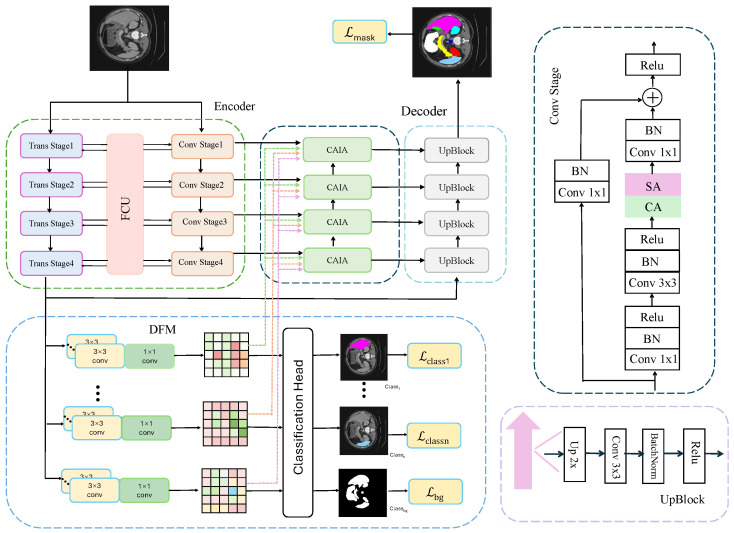
Overall framework of the proposed DCANet. Different colors represent the three core modules: FCU, DFM, and CAIA, as well as the convolution stages and upsampling blocks. Dashed/solid boxes and arrows denote intermediate/main components and data flows, respectively. The encoder–decoder architecture progressively integrates local and global features to achieve precise segmentation.

As illustrated in Figure 1, the proposed network adopts an encoder–decoder architecture with three core modules: FCU, DFM, and CAIA. FCU aligns and fuses convolutional and PVT features at each semantic level, enabling complementary modeling of local details and global context. The fused high-level features are then processed by DFM, which disentangles them into multi-class foreground and background features to enhance category discriminability and alleviate boundary ambiguity, optimized via a hybrid Cross-Entropy and Dice loss. Finally, CAIA performs category-aware multi-level feature aggregation using attention mechanisms, refining boundaries and improving inter-class separability, while the decoder progressively upsamples the aggregated features to produce precise segmentation maps.

### 3.2. Feature Coupling Unit (FCU)

**Figure 2:**
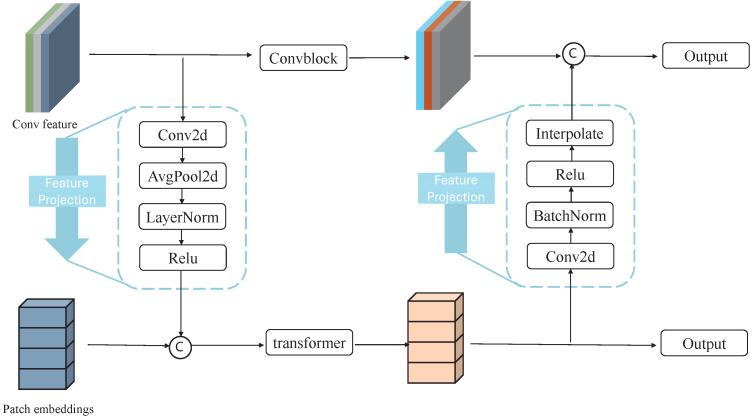
The structure of Feature Coupling Unit (FCU). Different colors represent different feature types (CNN features, Transformer features, projected features, and fused features). Dashed boxes/arrows indicate intermediate processing steps and data flows. FCU aligns heterogeneous features via projection and fuses them through concatenation to achieve effective integration of local and global representations.

As illustrated in Figure 2, the Feature Coupling Unit (FCU) is designed to effectively integrate local representations from the CNN branch with global contextual information from the Transformer branch, enabling deep interaction across heterogeneous encoding paradigms. Due to the inherent differences between CNN and Transformer features in terms of receptive fields, semantic abstraction levels, and statistical distributions, directly fusing these features may introduce semantic misalignment and limit their complementary benefits.

To address this issue, FCU first employs a feature projection operation to align CNN and Transformer features in both spatial resolution and semantic representation. Specifically, convolutional features extracted by the CNN branch are processed through a projection module consisting of Conv2D, AvgPool2D, LayerNorm, and GELU layers, which performs feature compression and normalization. This projection step mitigates distribution discrepancies caused by scale variations and different encoding mechanisms, mapping heterogeneous features into a unified embedding space.

After alignment, the projected CNN features are fused with the Transformer features via channel-wise concatenation. Unlike additive or weighted fusion strategies that implicitly assume semantic consistency between feature representations, concatenation preserves the complementary characteristics of local structural details and global contextual dependencies without enforcing strict semantic alignment. This design allows subsequent network layers to adaptively learn the relative importance of different feature channels, leading to more robust and stable feature fusion, which is particularly suitable for medical image segmentation tasks.

In addition, Transformer features undergo a reconstruction projection composed of Conv2D, BatchNorm, ReLU, and Interpolate operations to enhance high-level semantic representations and facilitate effective information propagation to the decoder. Each Transformer block takes the fused output from FCU together with token embeddings from the previous stage, enabling multi-level feature coupling and collaborative representation learning across the network.

### 3.3. Feature Decoupling Module (DFM)

The Feature Decoupling Module (DFM) is designed to explicitly disentangle the high-level features produced by the encoder into complementary representations of multi-class foregrounds and background, thereby enhancing category discriminability and mitigating semantic ambiguity. Specifically, the encoder output feature *f* is fed into a decoupling structure consisting of multiple parallel branches, each composed of several convolutional units to capture features at different semantic levels. Through this process, the model obtains a set of foreground feature maps $\left\{f_{1}^{fg},f_{2}^{fg},\ldots ,f_{k}^{fg}\right\}$ and a background feature map $f^{bg}$, jointly forming k+1 complementary semantic representations.

Subsequently, DFM employs auxiliary classification heads to predict these feature maps. Using a softmax operation $\mathrm{softmax}(f_{k}^{fg})$, the module generates the corresponding foreground masks $\left\{y_{1}^{fg},y_{2}^{fg},\ldots ,y_{k}^{fg}\right\}$ and the background mask $y^{bg}$. The pixel values of these masks are expected to be close to binary (0 or 1) and satisfy the complementary constraint between foreground and background, i.e., for any pixel index *i*, only one mask should be activated:

(1) $$y_{i,k}^{fg},y_{i}^{bg}\in \left\{0,1\right\}$$

(2) $$\sum_{k=1}^{n}y_{i,k}^{fg}+y_{i}^{bg}=1$$

Accordingly, we optimize the foreground masks $\left\{y_{k}^{fg}\right\}$ and the background mask $y^{bg}$ using a combination of cross-entropy (CE) [18] and Dice loss [19], encouraging them to approximate the ground-truth masks and their complementary regions (1−Mask), respectively. However, since small targets occupy only a tiny fraction of pixels, their contribution to the overall loss is often overshadowed by large targets. To address this issue, we introduce a dynamic weighting mechanism that adaptively adjusts the importance of each branch according to the pixel proportion of each foreground mask, defined as:

(3) $$\beta_{k}^{fg}=1-\sigma \left(\alpha \left(\frac{1}{N}\sum_{i=1}^{N}y_{i,k}^{fg}-\mu \right)\right)$$

where $\sigma \left(x\right)=\frac{1}{1+e^{-x}}$ is the Sigmoid function, α controls the sensitivity of the weight to changes in pixel proportion, *N* denotes the total number of pixels in the image, and $y_{i,k}^{fg}$ indicates the predicted foreground mask of class *k* at pixel *i*. The term μ represents the mean pixel proportion across all foreground categories and is defined as

(4) $$\mu =\frac{1}{K}\sum_{k=1}^{K}\frac{1}{N}\sum_{i=1}^{N}y_{i,k}^{fg}$$

This strategy assigns higher optimization weights to small objects (e.g., small organs or lesions) and reduces the weights for large objects. The resulting weight $\beta_{k}^{fg}$ is applied to the CE-Dice loss as

(5) $$\beta_{k}^{fg}L_{k},\;k=1,2,\ldots ,K,$$

effectively preventing small targets from being ignored during training.

Specifically, for each foreground category *k*, a weighted segmentation loss is formulated as

(6) $$L_{k}=L_{CE}\left(y_{k}^{fg},g_{k}\right)+L_{Dice}\left(y_{k}^{fg},g_{k}\right),$$

where $g_{k}$ denotes the corresponding ground-truth mask.

The overall loss of DFM is defined as

(7) $$L_{DFM}=\sum_{k=1}^{K}\beta_{k}^{fg}L_{k}+\lambda L_{bg},$$

where $L_{bg}$ represents the segmentation loss of the background branch and λ is a balancing coefficient.

In our experiments, the sensitivity parameter α in Equation (3) is empirically set to 5 according to the ablation study, and the balancing coefficient λ is fixed to 0.3 for all datasets.

In summary, DFM explicitly disentangles semantic features and incorporates a dynamic weighting strategy, which not only strengthens inter-foreground category discriminability but also significantly improves the modeling of small targets, providing a more reliable semantic foundation for subsequent decoding and category-aware aggregation.

### 3.4. Category-Aware Integration Aggregator (CAIA)

**Figure 3:**
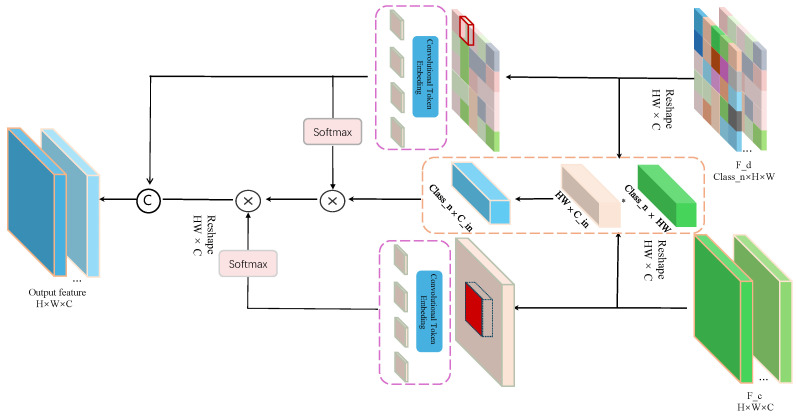
The structure of Category-Aware Integration Aggregator (CAIA). Different colors denote different feature types; dashed/solid boxes and arrows represent intermediate/final features and data flows. ∗ denotes matrix multiplication. H×W×C: feature map dimensions (height × width × channels).

The Category-Aware Integration Aggregator (CAIA) is designed to adaptively fuse multi-level features under the guidance of learned explicit category priors from the DFM module. As shown in Figure 3, unlike conventional fusion strategies that treat all features equally, CAIA introduces category-aware attention to explicitly model the relationship between category semantics and cross-level features, thereby enhancing inter-class discriminability and boundary sensitivity.

Specifically, encoder features $e_{1}$ to $e_{4}$ (after convolutional refinement) are concatenated with the previous layer’s output to form the fused feature map $F_{fuse}$. Meanwhile, the DFM module outputs foreground masks $y_{k}^{fg}$ (k=1,…,K) and a background mask $y^{bg}$, which are supervised by CE and Dice losses during training, ensuring that each pixel explicitly corresponds to a specific class or the background. These learned explicit category priors serve as interpretable spatial guidance and form the queries for CAIA to focus attention on relevant regions. Before attention computation, CAIA establishes contextual interactions between the category priors and the fused features:

(8) $$C_{ctx}={\left(F_{fuse}^{\prime }\right)}^{⊤}Y_{prior}^{\prime },$$

where $F_{fuse}^{\prime }$ and $Y_{prior}^{\prime }$ denote the reshaped fused features and multi-category prior masks from DFM, respectively. This operation captures semantic and spatial correlations between classes and feature locations, allowing attention updates to be guided by category semantics rather than relying solely on local context, enhancing cross-level feature discriminability and target focus.

Subsequently, the queries *Q* derived from the foreground masks $y_{k}^{fg}$ interact with keys *K* and values *V* generated from the context-enhanced fused features $C_{ctx}$:

(9) $$Q=\mathrm{Conv}\left(Y_{prior}^{fg}\right),\;K=\mathrm{Conv}\left(C_{ctx}\right),\;V=\mathrm{Conv}\left(C_{ctx}\right)$$

The attention computation is formulated as

(10) $$A=\mathrm{Softmax}\left(\frac{Q^{⊤}K}{C}\right),\;F_{att}=VA,$$

which adaptively emphasizes category-relevant regions while suppressing background, thereby enhancing inter-class separability and boundary sensitivity. Finally, the attention-enhanced feature $F_{att}$ is refined via a 1×1 convolution and fused with the decoder feature $e_{dec}$:

(11) $$F_{out}={\mathrm{Conv}}_{1\times 1}\left(F_{att}\right)+e_{dec}.$$

In summary, CAIA leverages learned explicit category priors $y_{k}^{fg}$ from DFM to guide attention-driven cross-level feature fusion, providing a principled mechanism for improved target focus, boundary refinement, and discriminative representation.

## 4. Experiments and Results

### 4.1. Datasets and Evaluation Metrics

Synapse multi-organ dataset: The Synapse multi-organ dataset consists of abdominal CT scans from 30 patients, totaling 3779 axial contrast-enhanced CT slices. Each image has a resolution of 512 × 512 pixels, and the dataset is provided in a 2D slice format, making it well-suited for multi-organ segmentation tasks. This dataset is widely used in medical image analysis research, particularly in deep learning-driven automatic segmentation tasks. Following the experimental setup of TransUNet [20], we randomly split the dataset into 18 cases for training (a total of 2212 axial slices) and the remaining 12 cases for validation. The dataset is precisely annotated with eight major anatomical structures, including the aorta, gallbladder, left and right kidneys, liver, pancreas, spleen, and stomach, providing high-quality reference data for training and evaluating medical image segmentation models. The main challenges of this dataset lie in the significant contrast differences between organs, the blurred boundaries of certain structures, and the large anatomical variations across different patients, making the segmentation task particularly challenging. ACDC Dataset: The ACDC (Automatic Cardiac Diagnosis Challenge) dataset consists of cardiac MRI images from 100 patients, with slice thicknesses ranging from 5 to 8 mm. It is specifically designed for cardiac structure segmentation tasks. Each scan includes annotations for three categories: the left ventricle, right ventricle, and myocardium. According to the experimental setup of TransUNet, we randomly split the dataset into 70 cases (a total of 1930 axial slices) for training, 10 cases for validation, and 20 cases for testing. The challenges of the ACDC dataset stem from the significant variations in cardiac morphology across different patients, as well as potential differences in tissue contrast caused by varying MRI scanning conditions, increasing the uncertainty of the segmentation task. Glas: The GlaS (Gland Segmentation) dataset [21] contains a total of 165 stained histological images of colorectal cancer tissue, with 85 images used for training and 80 for testing. MoNuSeg: The MoNuSeg (Multi-Organ Nuclei Segmentation) dataset [22] consists of 44 histological images from multiple organs, with 30 images used for training and 14 for testing.

Evaluation Metrics: In the experiments on the Synapse multi-organ dataset, we use the Dice coefficient and the 95% Hausdorff Distance (95HD) as evaluation metrics to measure segmentation accuracy and boundary approximation. For the ACDC dataset, the Dice coefficient is used to evaluate the segmentation performance of cardiac structures. For the GlaS and MoNuSeg datasets, both the Dice coefficient and Intersection over Union (IoU) are adopted to comprehensively assess the performance on gland and nuclei segmentation tasks. The Dice, 95HD, and IoU metrics are calculated using the following formulas:

(12) $$\begin{array}{c} DICE\left(X,Y\right)=\frac{2|X\cap Y|}{\left|X|+|Y\right|} \end{array}$$

Here, X and Y represent the sets of predicted labels and ground truth labels, respectively. The Dice coefficient ranges from 0 to 1.

(13) $$\begin{array}{c} HD\left(X,Y\right)=max\left\{d_{XY},d_{YX}\right\}=max\left\{\underset{x\in Xy\in Y}{maxmind(x,y)},\underset{y\in Yx\in X}{maxmind(x,y)}\right\} \end{array}$$

For the Hausdorff Distance (HD), X and Y represent the boundaries of the predicted and ground truth segmentation regions. The HD measures the maximum distance between these boundaries, where a smaller value indicates lower segmentation error at the boundaries and higher quality. To eliminate the impact of outliers and ensure stability, the 95% Hausdorff Distance is typically used, which considers the distance ranked in the top 95% (from smallest to largest) as the effective Hausdorff Distance.

(14) $$\begin{array}{c} IoU=\frac{TP}{TP+FP+FN} \end{array}$$

where TP denotes true positives (pixels correctly predicted as foreground), FP denotes false positives (pixels incorrectly predicted as foreground), and FN denotes false negatives (foreground pixels missed by the prediction). A higher IoU indicates better segmentation accuracy.

### 4.2. Implementation Details

**Figure 4:**
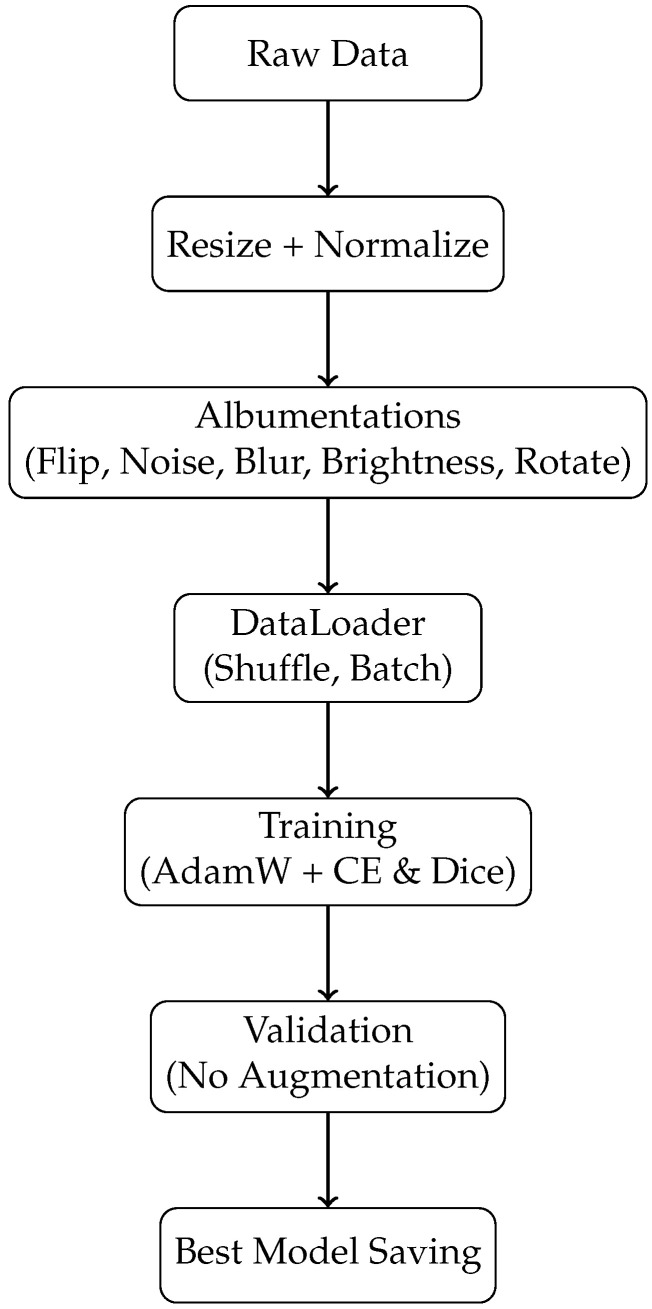
Overview of the proposed training and evaluation pipeline.

Our model was implemented using the PyTorch framework and trained on a single NVIDIA GeForce RTX 4060 GPU (NVIDIA Corporation, Santa Clara, CA, USA) with 12 GB of memory. An overview of the complete training and evaluation pipeline is illustrated in Figure 4. As shown in the figure, raw images from the Synapse, ACDC, GlaS, and MoNuSeg datasets were first resized to 224×224 pixels using bilinear interpolation and then normalized with dataset-specific mean and standard deviation values. During training, on-the-fly data augmentation was applied using the Albumentations library [23], including random horizontal and vertical flips, Gaussian blur with kernel sizes randomly selected between 1 and 3, Gaussian noise with variance ranging from 10.0 to 50.0, random brightness and contrast adjustments (brightness ±0.2, contrast ±0.3), and random shift, scale, and rotation operations (shift ±0.2, scale ±0.5, rotation $\pm 40^{\circ }$). Each augmentation was applied with a probability of 0.5. The augmented images were then loaded using the PyTorch (version 2.10.0) DataLoader with shuffling enabled and grouped into mini-batches for network training.

The maximum number of training epochs was set to 150 with a batch size of 12, and the initial learning rate was set to 0.001. We adopted the AdamW optimizer [24] for network optimization, where the first- and second-order moment coefficients were set to $\beta_{1}=0.9$ and $\beta_{2}=0.999$, respectively. The numerical stability constant was $\epsilon =1\times 10^{-8}$, and the weight decay was set to $1\times 10^{-4}$. A hybrid loss function combining Cross-Entropy Loss and Dice Loss was employed to mitigate class imbalance and improve segmentation accuracy.

For validation, images were deterministically resized and normalized without any data augmentation, as depicted in Figure 4. Model parameters were initialized using the default PyTorch initialization scheme. To ensure reproducibility, random seeds for Python, NumPy, and PyTorch were fixed, and deterministic cuDNN settings were enabled. The model achieving the best validation performance was saved for final evaluation.

### 4.3. Loss Function Design

In multi-organ medical image segmentation, it is crucial to simultaneously ensure pixel-wise classification accuracy and region-level overlap consistency. To this end, we adopt a weighted composite loss function that combines the Cross-Entropy(*CE*) loss and the Dice loss. The overall loss function is defined as

(15) $$L_{CE-Dice}=\lambda_{1}L_{CE}+\lambda_{2}L_{Dice},$$

where the Cross-Entropy loss is formulated as

(16) $$L_{CE}=-\frac{1}{N}\sum_{i=1}^{N}\left[y_{i}\log\left(p_{i}\right)+\left(1-y_{i}\right)\log\left(1-p_{i}\right)\right],$$

and the Dice loss is defined as

(17) $$L_{Dice}=1-\frac{2\sum_{i=1}^{N}p_{i}y_{i}}{\sum_{i=1}^{N}p_{i}+\sum_{i=1}^{N}y_{i}}.$$

Here, $p_{i}$ denotes the predicted probability for pixel *i*, $y_{i}$ represents the corresponding ground-truth label, and *N* is the total number of pixels. The Cross-Entropy loss provides stable pixel-level supervision and facilitates efficient optimization, while the Dice loss directly optimizes the overlap between predicted regions and ground truth, effectively alleviating class imbalance and enhancing the segmentation of small or irregular anatomical structures.

Through extensive empirical evaluation, we find that setting $\lambda_{1}=0.4$ and $\lambda_{2}=0.6$ yields the best overall segmentation performance. Therefore, this weighting configuration is adopted in all experiments.

### 4.4. Experimental Results on the Synapse Dataset

The proposed DCANet achieves competitive performance on the Synapse multi-organ segmentation dataset in terms of both Dice score and HD95. As shown in Table 1, all results are averaged over five independent runs with fixed training and testing splits, yielding a mean Dice score of 84.80% ± 0.67% and an average HD95 of 14.73 ± 0.47 mm. Compared with VM-UNet, DCANet improves the Dice score by 3.72% and reduces the HD95 by 4.48 mm, indicating improved region overlap and boundary accuracy.

An organ-wise analysis reveals both the strengths and limitations of DCANet. For large and elongated organs such as the pancreas and spleen, DCANet shows clear improvements, benefiting from enhanced global contextual modeling. For smaller or low-contrast organs, including the gallbladder and kidneys, the performance gains are more moderate, indicating that DCANet still faces challenges in extreme small-target segmentation, a limitation shared by most CNN- and Transformer-based methods.

In addition to CNN- and Transformer-based approaches, we further compare DCANet with recent fusion-based models such as MFSE-Net. While fusion-based methods improve robustness by integrating multi-scale or multi-source features, they often rely on implicit feature aggregation and may suffer from feature misalignment. By comparison, DCANet employs a collaborative encoder that explicitly models cross-branch interactions, enabling more effective fusion of local structural and global semantic information. As a result, DCANet achieves superior performance among fusion-based methods, although this explicit interaction mechanism introduces additional computational complexity compared with simpler fusion strategies.

**Figure 5:**
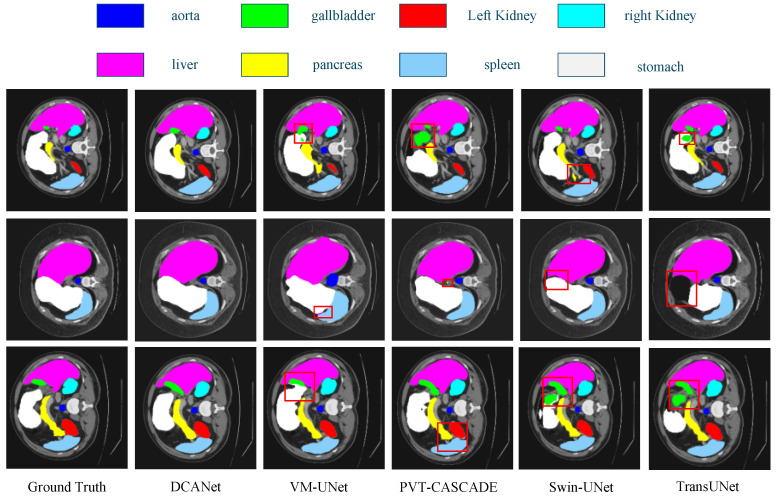
Qualitative comparison on the Synapse dataset. The red boxes indicate the failure cases of other methods, where our method achieves correct segmentation.

Figure 5 provides qualitative comparisons between DCANet and representative methods, including VM-UNet, PVT-CASCADE, SwinUNet, and TransUNet. DCANet generates more continuous and anatomically consistent segmentation boundaries for multiple organs, such as the aorta, liver, pancreas, and spleen. Compared with these methods, other approaches occasionally produce fragmented predictions or boundary leakage in regions with ambiguous contrast or irregular morphology. These visual results demonstrate that DCANet effectively preserves structural continuity and reduces misclassified regions in challenging scenarios.

It is worth noting that DCANet introduces additional computational overhead due to multi-branch feature coupling and category-aware modeling. Although this overhead remains acceptable in practical settings, future work will explore more efficient designs to further reduce complexity while maintaining segmentation accuracy.

### 4.5. Experimental Results on ACDC Dataset

Our proposed DCANet demonstrates strong segmentation performance on the ACDC dataset, with all results averaged over five independent experiments using fixed training and testing splits. As reported in Table 2, DCANet achieves an average Dice score of 94.07% ± 0.41% and an average HD95 of 1.24 ± 0.23, outperforming the recent PVT-CASCADE method by 2.61% in terms of Dice score. For individual cardiac structures, DCANet attains Dice scores of 93.78% for the right ventricle (RV), 91.72% for the myocardium (Myo), and 96.72% for the left ventricle (LV), indicating consistent improvements across different anatomical regions.

Beyond CNN- and Transformer-based architectures, we also compare DCANet with recent fusion-based methods such as MFSE-Net. While fusion-based approaches improve robustness by integrating multi-scale or multi-source features, they often rely on implicit feature aggregation. By comparison, DCANet employs an explicit feature fusion and category-aware modeling strategy, achieving superior performance not only over conventional architectures but also among fusion-based methods.

Compared with CNN-based models such as R50 + UNet and AttnUNet, DCANet benefits from enhanced global contextual modeling, which helps reduce local ambiguity in cardiac structures with complex shapes. Transformer-based methods such as TransUNet, SwinUNet, and MISSFormer emphasize global representations but may compromise boundary precision, particularly in thin or low-contrast regions. By integrating local structural information through multi-level feature fusion, DCANet provides a more balanced representation of global context and fine-grained boundaries.

Despite these advantages, DCANet still exhibits certain limitations. The performance gains for thin structures such as the myocardium are relatively modest compared with those for larger regions, indicating ongoing challenges in segmenting extremely thin or low-contrast tissues. In addition, the collaborative encoder and explicit fusion mechanisms introduce increased computational complexity, which may limit deployment in resource-constrained clinical settings. Addressing these limitations through more efficient fusion strategies and lightweight architectures will be explored in future work.

**Figure 6:**
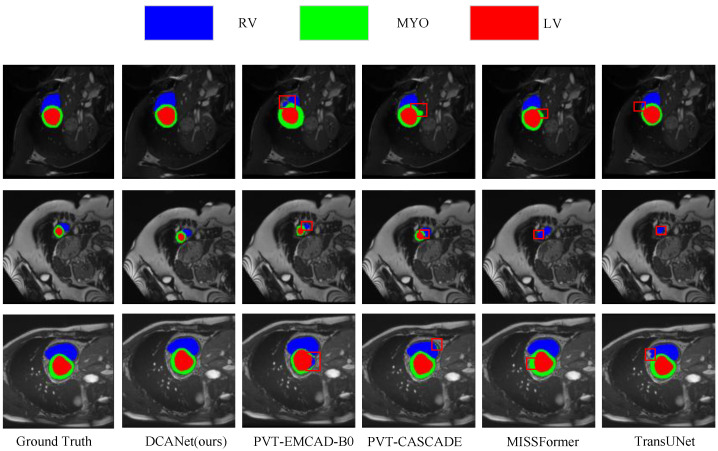
Qualitative comparison of different methods on the ACDC dataset. The red boxes indicate the failure cases of other methods, where our method achieves correct segmentation.

Figure 6 presents qualitative comparisons among DCANet, PVT-EMCAD-B0, PVT-CASCADE, MISSFormer, and SwinUNet. DCANet produces more continuous and anatomically consistent contours, particularly for the RV and Myo, where competing methods tend to exhibit boundary leakage, fragmented predictions, or over- and under-segmentation. These qualitative observations are consistent with the quantitative results, further confirming that DCANet achieves a favorable trade-off between global context modeling and precise boundary delineation for cardiac structure segmentation.

### 4.6. Experimental Results on the GlaS and MoNuSeg Datasets

The quantitative results of DCANet on the GlaS and MoNuSeg datasets are summarized in Table 3. All reported results are averaged over five independent runs using fixed training and testing splits and are presented as mean ± standard deviation. On the GlaS dataset, DCANet achieves Dice and IoU scores of 94.60 ± 0.6% and 89.48 ± 0.8%, respectively, outperforming most competing methods. The GlaS dataset focuses on gland segmentation with irregular shapes and varying scales, where accurate boundary delineation and structural continuity are critical. Compared with CNN-based methods that primarily rely on local features, DCANet benefits from the integration of global contextual information, enabling more coherent modeling of glandular structures. Meanwhile, compared with Transformer-based approaches that emphasize global representations, DCANet preserves fine-grained boundary details through multi-level feature fusion.

On the MoNuSeg dataset, which is characterized by densely packed nuclei and high intra-class variability, DCANet achieves the highest IoU score of 66.47 ± 0.5% and a competitive Dice score of 79.85 ± 0.7%. In this scenario, methods relying solely on global context may suffer from nucleus adhesion, while purely local feature-based models often produce fragmented predictions. By explicitly combining local structural cues with global semantic dependencies, DCANet demonstrates improved discriminative capability for closely adjacent nuclei and effectively reduces segmentation ambiguity.

Overall, the experimental results on both datasets indicate that DCANet exhibits strong generalization ability across different histopathological segmentation tasks. By jointly modeling local details and global context and incorporating category-aware feature interactions, the proposed method effectively handles both gland-level and nucleus-level segmentation challenges, maintaining high accuracy and structural consistency across diverse biomedical image segmentation scenarios, although it may still struggle with extremely small or highly overlapping structures and requires relatively high computational resources.

**Figure 7:**
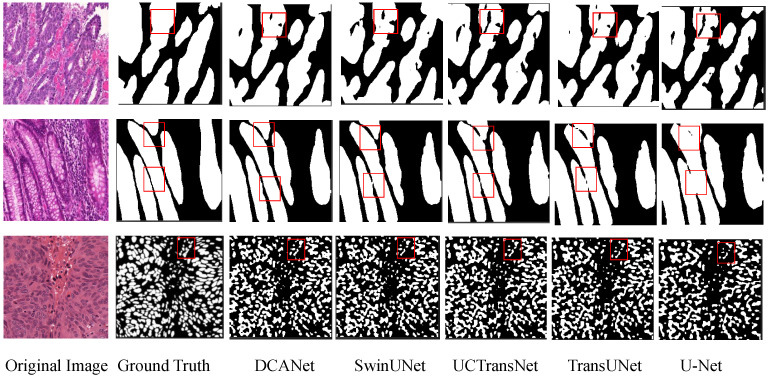
Qualitative comparison of different methods on the GlaS and MoNuSeg datasets. The red boxes highlight the regions where our method achieves the best segmentation results compared to others.

Figure 7 presents qualitative comparisons among DCANet, SwinUNet, UCTransNet, TransUNet, and U-Net on the GlaS and MoNuSeg datasets. On GlaS, DCANet produces clearer gland boundaries and more continuous structures in regions with complex morphology, whereas other methods tend to generate blurred boundaries or locally missing predictions. On MoNuSeg, DCANet shows stronger separation ability for densely clustered nuclei, reducing adhesion artifacts and yielding smoother contours. These qualitative observations are consistent with the quantitative results, further validating the effectiveness of DCANet in complex histopathological segmentation tasks.

### 4.7. Failure Case Analysis

**Figure 8:**
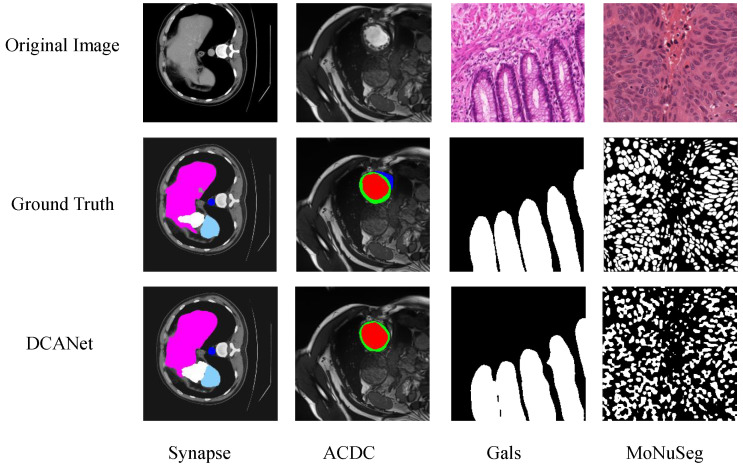
Qualitative failure case analysis of DCANet on the Synapse, ACDC, GlaS, and MoNuSeg datasets.

Despite the overall strong performance of DCANet, several challenging scenarios remain difficult to handle, as illustrated in Figure 8. On the Synapse dataset, DCANet may produce slight under-segmentation or boundary inaccuracies when multiple abdominal organs are tightly adjacent and exhibit low contrast, where ambiguous organ boundaries hinder precise separation. For the ACDC dataset, failure cases are mainly observed in slices with extreme cardiac shape variations or blurred myocardial borders, in which large inter-patient anatomical differences can still lead to minor mis-segmentation. On the GlaS dataset, errors typically occur in gland regions with highly irregular morphologies or thin elongated structures, where fine-grained details are prone to partial omission during feature aggregation. Similarly, on the MoNuSeg dataset, DCANet may struggle with densely clustered nuclei, resulting in fragmented or merged predictions due to severe spatial overlap and high structural similarity among instances. These failure cases suggest that DCANet, like other learning-based segmentation models, remains sensitive to boundary ambiguity, severe shape variation, and extremely small or densely packed targets, indicating potential directions for future improvement.

### 4.8. Ablation Study

#### 4.8.1. Ablation Study on Different Module Designs

Table 4 presents the ablation results of different model variants. Variant (a) is the baseline model, where features from the convolutional encoder and the Transformer encoder are simply concatenated and fed into a convolutional decoder for segmentation. Variant (b) introduces the Feature Coupling Unit (FCU), which aligns and fuses convolutional and Transformer features before decoding, resulting in a clear performance improvement across all datasets. Variant (c) further incorporates the Decoupled Feature Module (DFM), which decomposes high-level features into foreground and background representations and integrates them into the decoder input, leading to additional gains in both region accuracy and boundary quality. Variant (d) corresponds to the full DCANet, where the Category-Aware Integration Aggregator (CAIA) is added to guide feature fusion with category-aware attention. The complete DCANet achieves the best performance on all four datasets, obtaining Dice scores of 84.80% on Synapse, 94.07% on ACDC, 94.60% on GlaS, and 79.85% on MoNuSeg, along with consistently improved boundary-related metrics, demonstrating the complementary effectiveness of FCU, DFM, and CAIA.

**Figure 9:**
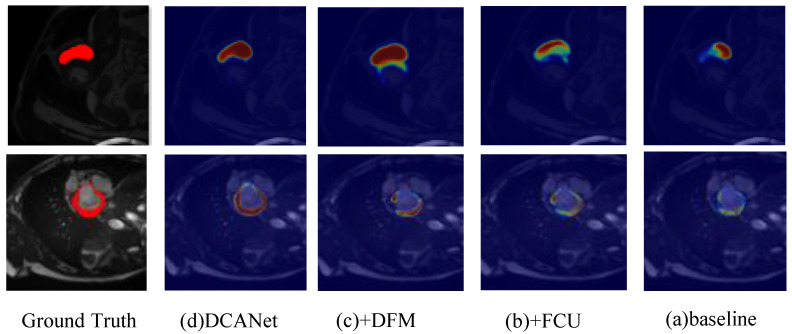
Qualitative comparison of activation heatmaps for different ablation variants on the ACDC dataset. (**a**) Baseline model with direct concatenation of convolutional and Transformer features; (**b**) Introduction of FCU for improved feature alignment; (**c**) Addition of DFM for foreground/background decoupling; (**d**) Full DCANet with all components (FCU, DFM, and CAIA).

Figure 9 visualizes the activation heatmaps of different ablation variants on the ACDC dataset. Compared with the ground truth, the baseline model (a), which directly concatenates convolutional and Transformer features, shows dispersed and imprecise activations with noticeable responses in non-target regions. After introducing FCU in (b), the activation becomes more concentrated, indicating that aligned fusion of local and global features helps the network better capture the target structure. Further incorporating DFM in (c) leads to more compact and boundary-aware activations, suggesting that foreground/background decoupling enables the model to focus on anatomically relevant regions while suppressing background interference. The full DCANet produces the most accurate and well-localized activation maps, closely matching the ground truth with clear boundaries and minimal background responses, demonstrating the complementary effectiveness of FCU, DFM, and CAIA in guiding discriminative feature learning.

**Figure 10:**
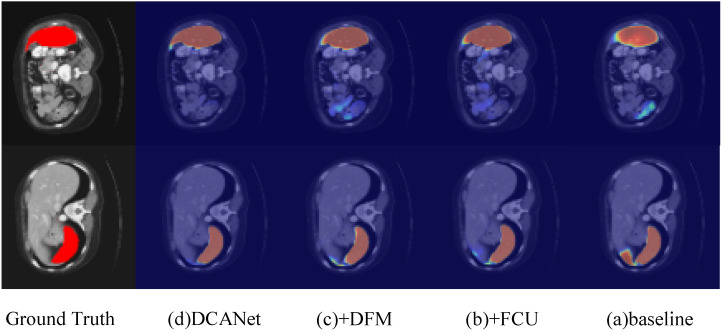
Qualitative comparison of activation heatmaps for different ablation variants on the Synapse dataset. (**a**) Baseline; (**b**) +FCU; (**c**) +FCU+DFM; (**d**) Full DCANet (FCU+DFM+CAIA).

Figure 10 illustrates the activation heatmaps of different ablation variants on the Synapse dataset. Compared with the ground truth, the baseline model (a), which directly concatenates convolutional and Transformer features, exhibits diffuse activations and noticeable responses in non-target regions, especially around complex abdominal structures. After introducing FCU in (b), the activation distribution becomes more concentrated, indicating improved alignment and fusion of local and global features. Further adding DFM in (c) results in more compact and anatomically consistent activations, particularly along organ boundaries, demonstrating that foreground/background decoupling effectively suppresses irrelevant background responses. The full DCANet produces the most accurate and well-localized heatmaps, closely matching the ground truth with clearer boundaries and reduced ambiguity, highlighting the complementary roles of FCU, DFM, and CAIA in enhancing feature discriminability for complex multi-organ segmentation.

#### 4.8.2. Ablation Study on FCU

Unlike conventional attention or fusion modules such as SE, CBAM, ECA, and AFF, which mainly perform channel- or spatial-wise re-weighting of feature maps, the proposed Feature Coupling Unit (FCU) explicitly fuses convolutional and Transformer features. Convolutional features capture fine-grained local structural information, while Transformer features encode long-range contextual dependencies. FCU first aligns these heterogeneous features via spatial and semantic projection, then concatenates them along the channel dimension to produce a unified representation at each semantic level.

This explicit fusion allows the network to simultaneously leverage local and global information, enhancing the discriminative ability for different classes and improving boundary delineation, particularly for small or complex anatomical structures. Compared with these modules, other attention/fusion modules lack such direct cross-modal feature integration, limiting their effectiveness in modeling long-range dependencies and fine-grained details. As demonstrated in Table 5, FCU consistently outperforms these modules across Synapse, ACDC, GlaS, and MoNuSeg datasets, validating the advantage of explicit convolution-Transformer fusion.

#### 4.8.3. Ablation Study of the Hyperparameter α in DFM

To investigate the influence of the hyperparameter α in the proposed dynamic weighting mechanism, we conduct a sensitivity analysis by varying α in {0,1,2,5,10}. When α=0, the dynamic weighting strategy is disabled, serving as a baseline. All experiments are conducted under identical training settings to ensure fair comparison.

As shown in Table 6, the segmentation performance gradually improves when α increases from 0 to 5, indicating that moderate dynamic weighting effectively enhances the modeling of small targets. However, further increasing α to 10 leads to a slight performance degradation, suggesting that overly aggressive weighting may introduce instability. Therefore, α=5 is adopted in all experiments.

#### 4.8.4. Ablation Study on Loss Weighting

To analyze the impact of different loss weighting strategies, we conduct an ablation study on the weighting coefficients $\left(\lambda_{1},\lambda_{2}\right)$ of the combined Cross-Entropy and Dice loss. Table 7 reports the segmentation performance on the Synapse and ACDC datasets under different weight configurations.

As shown in Table 7, assigning a higher weight to the Dice loss generally leads to improved segmentation performance. When $\lambda_{1}=0.4$ and $\lambda_{2}=0.6$, DCANet achieves the best overall results, obtaining the highest Dice scores and the lowest HD95 on the Synapse dataset, as well as the highest Dice score on the ACDC dataset. This configuration provides a balanced optimization between pixel-level classification accuracy and region-level overlap consistency.

By comparison, configurations that place greater emphasis on the Cross-Entropy loss (e.g., $\lambda_{1}=0.6$, $\lambda_{2}=0.4$) yield inferior performance, particularly in boundary accuracy, as indicated by higher HD95 values. This observation suggests that Dice loss plays a more critical role in alleviating class imbalance and preserving anatomical structure continuity in multi-organ segmentation tasks.

Based on these results, the loss weighting $\left(\lambda_{1},\lambda_{2}\right)=\left(0.4,0.6\right)$ is adopted as the default configuration in all experiments.

## 5. Conclusions

In this paper, we propose DCANet for medical image segmentation, aiming to improve the delineation of complex anatomical structures with ambiguous boundaries and large appearance variations. By jointly leveraging local structural cues, global contextual information, and category-aware representations, DCANet achieves accurate and robust segmentation across diverse organ types and imaging scenarios.

Extensive experiments on four public benchmarks, including Synapse, ACDC, GlaS, and MoNuSeg, demonstrate that DCANet consistently outperforms state-of-the-art methods in terms of segmentation accuracy and boundary quality. The superior performance on both large and small structures indicates strong generalization capability and robustness across different medical image segmentation tasks.

Nevertheless, DCANet may still encounter challenges in extremely difficult cases, such as those with severely blurred boundaries, highly imbalanced small targets, or dense clustering. In addition, the incorporation of multi-branch feature fusion and category-aware interactions introduces additional model complexity compared with lightweight approaches. Future work will focus on improving computational efficiency and exploring adaptive strategies to further enhance segmentation performance under challenging conditions.

## Figures and Tables

**Table 1:** Results of multi-organ segmentation on the Synapse dataset. Arch. Type denotes the primary architectural modeling strategy. Fusion indicates methods that explicitly integrate features from multiple sources or scales. Organ abbreviations: GB (Gallbladder), KL (Left Kidney), KR (Right Kidney), PC (Pancreas), SP (Spleen), SM (Stomach). ↑ indicates higher values are better, while ↓ indicates lower values are better.

Architectures	Arch. Type	Average		Aorta	GB	KL	KR	Liver	PC	SP	SM
		DICE ↑	HD95 ↓								
UNet [10]	CNN	70.11	44.69	84.00	56.70	72.41	62.64	86.98	48.73	81.48	67.96
AttnUNet [2]	CNN + Attn	71.70	34.47	82.61	61.94	76.07	70.42	87.54	46.70	80.67	67.66
R50 + UNet [20]	CNN	74.68	36.87	84.18	62.84	79.19	71.29	93.35	48.23	84.41	73.92
R50 + AttnUNet [20]	CNN + Attn	75.57	36.97	55.92	63.91	79.20	72.71	93.56	49.37	87.19	74.95
SSFormerPVT [25]	Trans	78.01	25.72	82.78	63.74	80.72	78.11	93.53	61.53	87.07	76.61
PolypPVT [26]	Trans	78.08	25.61	82.34	66.14	81.21	73.78	94.37	59.34	88.05	79.40
TFCNs [27]	Multi-scale	75.63	30.63	88.23	59.18	80.99	73.12	92.02	54.24	88.36	68.90
TransUNet [20]	CNN + Trans	77.48	31.69	87.23	63.13	81.87	77.02	94.08	55.86	85.08	75.62
SwinUNet [28]	Trans	79.13	21.55	85.47	66.53	83.28	79.61	94.29	56.58	90.66	76.60
HiFormer-L [29]	CNN + Trans	80.69	19.14	87.03	68.61	84.23	78.37	94.07	60.77	90.44	82.03
PVT-CASCADE [30]	Multi-scale	81.06	20.23	83.01	70.59	82.23	80.37	94.08	64.43	90.10	83.69
MFSE-Net [31]	Fusion	81.20	19.71	85.90	68.84	85.76	81.96	94.67	59.87	90.32	82.26
VM-UNet [32]	Mamba	81.08	19.21	86.40	69.41	86.16	82.76	94.17	58.80	89.51	81.40
DCANet (Ours)	Fusion	84.80	14.73	88.77	72.81	89.08	85.50	95.15	69.85	93.36	83.92
Improve VM-UNet	–	3.72	4.48	2.37	3.40	2.92	2.74	0.98	11.05	3.85	2.52

**Table 2:** Results of cardiac structure segmentation on the ACDC dataset. Arch. Type denotes the primary architectural modeling strategy, where Fusion indicates methods that explicitly integrate features from multiple sources or representations. All results of DCANet are reported as mean ± standard deviation over five independent runs, while the results of other methods are taken from their original publications. Organ abbreviations: RV (Right Ventricle), Myo (Myocardium), LV (Left Ventricle). Higher Dice and lower HD95 indicate better segmentation performance.

Architectures	Arch. Type	Avg DICE	HD95	RV	Myo	LV
R50 + UNet [20]	CNN	87.55	1.51	87.10	80.63	94.92
R50 + AttnUNet [20]	CNN + Attn	86.75	1.53	87.58	79.20	93.47
ViT + CUP [20]	Trans	81.45	1.62	81.46	70.71	92.18
R50 + ViT + CUP [20]	CNN + Trans	87.57	1.46	86.07	81.88	94.75
TransUNet [20]	CNN + Trans	89.71	1.08	88.86	84.53	95.73
SwinUNet [28]	Trans	90.00	1.32	88.55	85.62	95.83
MISSFormer [33]	Trans	90.86	2.32	89.55	88.04	94.99
PVT-CASCADE [30]	Multi-scale	91.46	1.35	88.90	89.97	95.50
PVT-EMCAD-B0 [34]	Multi-scale	91.34	1.38	89.37	88.99	95.65
MFSE-Net [31]	Fusion	91.80	1.29	89.57	89.37	96.45
DCANet (Ours)	Fusion	94.07	1.24	93.78	91.72	96.72

**Table 3:** Quantitative results on the GlaS and MoNuSeg datasets. All results are reported as mean ± standard deviation over five independent runs using fixed data splits. Dice and IoU metrics indicate segmentation accuracy, with higher values representing better performance.

Architecture	GlaS		MoNuSeg	
	Dice	IoU	Dice	IoU
U-Net [35]	85.45 ± 1.3	74.78 ± 1.7	76.45 ± 2.6	62.86 ± 3.0
UNet++ [35]	87.56 ± 1.2	79.13 ± 1.7	77.01 ± 2.1	63.04 ± 2.5
AttUNet [35]	88.80 ± 1.1	80.69 ± 1.7	76.67 ± 1.1	63.47 ± 1.2
TransUNet [35]	88.40 ± 0.7	80.40 ± 1.0	78.53 ± 1.1	65.05 ± 1.3
MedT [35]	85.92 ± 2.9	75.47 ± 3.5	77.46 ± 2.4	63.37 ± 3.1
Swin-Unet [35]	89.58 ± 0.6	82.06 ± 0.7	77.69 ± 0.9	63.77 ± 1.2
UCTransNet [35]	90.18 ± 0.7	82.96 ± 1.1	79.08 ± 0.7	65.50 ± 0.9
PAG-TransYNet [36]	94.20 ± 0.55	89.29 ± 0.91	79.62 ± 0.7	66.31 ± 0.6
DCANet (Ours)	94.60 ± 0.6	89.48 ± 0.8	79.85 ± 0.7	66.47 ± 0.5

**Table 4:** Ablation study of different modules on four datasets (Synapse, ACDC, GlaS, MoNuSeg).

Index	FCU	DFM	CAIA	Synapse		ACDC		GlaS		MoNuSeg	
				Dice	HD95	Dice	HD95	Dice	IoU	Dice	IoU
(a)	×	×	×	70.11	44.69	85.75	1.43	85.45	74.78	76.45	62.86
(b)	✓	×	×	82.62	17.83	91.72	1.58	91.45	86.81	77.54	64.67
(c)	✓	✓	×	83.24	16.21	92.35	1.36	92.74	87.68	78.65	65.15
(d)	✓	✓	✓	84.80	14.73	94.07	1.24	94.60	89.48	79.85	66.47

Note: × indicates the module is not used; ✓ indicates the module is used.

**Table 5:** Ablation study on different fusion modules.

Fusion Module	Synapse		ACDC	GlaS		MoNuSeg	
	Dice (%)	HD95	Dice (%)	Dice (%)	IoU (%)	Dice (%)	IoU (%)
SE [37]	83.10	16.40	91.90	91.80	87.00	77.80	65.50
CBAM [38]	83.45	16.10	92.05	92.10	87.35	78.05	65.80
ECA [39]	83.57	15.98	92.15	92.25	87.50	78.23	65.94
AFF [40]	83.70	15.86	92.28	92.38	87.65	78.41	66.05
FCU (Ours)	84.80	14.73	94.07	94.60	89.48	79.85	66.47

**Table 6:** Sensitivity analysis of hyperparameter α in DFM.

α	Synapse		ACDC		GlaS		MoNuSeg	
	Dice (%)	HD95	Dice (%)	HD95	Dice (%)	IoU (%)	Dice (%)	IoU (%)
0	83.97	15.23	91.47	1.59	92.11	87.63	77.52	63.91
1	84.32	14.97	91.95	1.47	93.18	88.92	78.88	65.52
2	84.51	14.84	92.48	1.41	94.60	89.48	79.53	65.83
5	84.80	14.73	94.07	1.24	93.79	88.96	79.85	66.47
10	84.12	14.91	92.71	1.38	93.42	88.64	78.79	65.12

**Table 7:** Ablation study on different loss weight configurations.

($\lambda_{1},\lambda_{2}$)	Synapse Dice (%)	Synapse HD95 (mm)	ACDC Dice (%)	ACDC HD95 (mm)
(0.6, 0.4)	84.06	15.45	93.24	1.37
(0.5, 0.5)	84.38	15.06	93.59	1.32
(0.4, 0.6)	84.80	14.73	94.07	1.24
(0.3, 0.7)	84.25	15.34	93.76	1.40

## Data Availability

The datasets used and analyzed during the current study are available from the corresponding author on reasonable request.

## References

[1] Zhou Z., Rahman Siddiquee M.M., Tajbakhsh N., Liang J. (2018). Unet++: A nested u-net architecture for medical image segmentation. Proceedings of the Deep Learning in Medical Image Analysis and Multimodal Learning for Clinical Decision Support: 4th International Workshop, DLMIA 2018, and 8th International Workshop, ML-CDS 2018, Conjunction with MICCAI 2018, Granada, Spain, 20 September 2018.

[2] Oktay O., Schlemper J., Folgoc L.L., Lee M., Heinrich M., Misawa K., Mori K., McDonagh S., Hammerla N.Y., Kainz B. (2018). Attention u-net: Learning where to look for the pancreas. arXiv.

[3] Xiao X., Lian S., Luo Z., Li S. (2018). Weighted res-unet for high-quality retina vessel segmentation. Proceedings of the 2018 9th International Conference on Information Technology in Medicine and Education (ITME), Hangzhou, China, 19–21 October 2018.

[4] Fan D.P., Ji G.P., Zhou T., Chen G., Fu H., Shen J., Shao L. (2020). Pranet: Parallel reverse attention network for polyp segmentation. Proceedings of the International Conference on Medical Image Computing and Computer-Assisted Intervention, Lima, Peru, 4–8 October 2020.

[5] Zheng J., Liu H., Feng Y., Xu J., Zhao L. (2023). CASF-Net: Cross-attention and cross-scale fusion network for medical image segmentation. Comput. Methods Programs Biomed..

[6] Peng C., Myronenko A., Hatamizadeh A., Nath V., Siddiquee M.M.R., He Y., Xu D., Chellappa R., Yang D. Hypersegnas: Bridging one-shot neural architecture search with 3d medical image segmentation using hypernet. Proceedings of the IEEE/CVF Conference on Computer Vision and Pattern Recognition.

[7] Huang Z., Wang Z., Yang Z., Gu L. (2022). Adwu-net: Adaptive depth and width u-net for medical image segmentation by differentiable neural architecture search. Proceedings of the International Conference on Medical Imaging with Deep Learning, Zurich, Switzerland, 6–8 July 2022.

[8] Karimi D., Salcudean S.E. (2019). Reducing the hausdorff distance in medical image segmentation with convolutional neural networks. IEEE Trans. Med. Imaging.

[9] Kervadec H., Bouchtiba J., Desrosiers C., Granger E., Dolz J., Ayed I.B. (2019). Boundary loss for highly unbalanced segmentation. Proceedings of the International Conference on Medical Imaging with Deep Learning, London, UK, 8–10 July 2019.

[10] Ronneberger O., Fischer P., Brox T. (2015). U-net: Convolutional networks for biomedical image segmentation. Proceedings of the Medical Image Computing and Computer-Assisted Intervention–MICCAI 2015: 18th International Conference, Munich, Germany, 5–9 October 2015.

[11] Li X., Chen H., Qi X., Dou Q., Fu C.W., Heng P.A. (2018). H-DenseUNet: Hybrid densely connected UNet for liver and tumor segmentation from CT volumes. IEEE Trans. Med. Imaging.

[12] Graham B., El-Nouby A., Touvron H., Stock P., Joulin A., Jégou H., Douze M. Levit: A vision transformer in convnet’s clothing for faster inference. Proceedings of the IEEE/CVF International Conference on Computer Vision.

[13] Liu Z., Lin Y., Cao Y., Hu H., Wei Y., Zhang Z., Lin S., Guo B. Swin transformer: Hierarchical vision transformer using shifted windows. Proceedings of the IEEE/CVF International Conference on Computer Vision.

[14] Wang W., Xie E., Li X., Fan D.P., Song K., Liang D., Lu T., Luo P., Shao L. Pyramid vision transformer: A versatile backbone for dense prediction without convolutions. Proceedings of the IEEE/CVF International Conference on Computer Vision.

[15] Xie E., Wang W., Yu Z., Anandkumar A., Alvarez J.M., Luo P. (2021). SegFormer: Simple and efficient design for semantic segmentation with transformers. Adv. Neural Inf. Process. Syst..

[16] Zhou T., Zhou Y., He K., Gong C., Yang J., Fu H., Shen D. (2023). Cross-level feature aggregation network for polyp segmentation. Pattern Recognit..

[17] Bui N.T., Hoang D.H., Nguyen Q.T., Tran M.T., Le N. Meganet: Multi-scale edge-guided attention network for weak boundary polyp segmentation. Proceedings of the IEEE/CVF Winter Conference on Applications of Computer Vision.

[18] Mao A., Mohri M., Zhong Y. (2023). Cross-entropy loss functions: Theoretical analysis and applications. Proceedings of the International Conference on Machine Learning, Honolulu, Hawaii, USA, 23–29 July 2023.

[19] Li X., Sun X., Meng Y., Liang J., Wu F., Li J. Dice loss for data-imbalanced NLP tasks. Proceedings of the 58th Annual Meeting of the Association for Computational Linguistics.

[20] Chen J., Lu Y., Yu Q., Luo X., Adeli E., Wang Y., Lu L., Yuille A.L., Zhou Y. (2021). Transunet: Transformers make strong encoders for medical image segmentation. arXiv.

[21] Sirinukunwattana K., Pluim J.P., Chen H., Qi X., Heng P.A., Guo Y.B., Wang L.Y., Matuszewski B.J., Bruni E., Sanchez U. (2017). Gland segmentation in colon histology images: The glas challenge contest. Med. Image Anal..

[22] Kumar N., Verma R., Anand D., Zhou Y., Onder O.F., Tsougenis E., Chen H., Heng P.A., Li J., Hu Z. (2019). A multi-organ nucleus segmentation challenge. IEEE Trans. Med. Imaging.

[23] Buslaev A., Iglovikov V.I., Khvedchenya E., Parinov A., Druzhinin M., Kalinin A.A. (2020). Albumentations: Fast and flexible image augmentations. Information.

[24] Loshchilov I. (2017). Decoupled weight decay regularization. arXiv.

[25] Wang J., Huang Q., Tang F., Meng J., Su J., Song S. (2022). Stepwise feature fusion: Local guides global. Proceedings of the International Conference on Medical Image Computing and Computer-Assisted Intervention, Singapore, 18–22 September 2022.

[26] Dong B., Wang W., Fan D.P., Li J., Fu H., Shao L. (2021). Polyp-pvt: Polyp segmentation with pyramid vision transformers. arXiv.

[27] Li Z., Li D., Xu C., Wang W., Hong Q., Li Q., Tian J. (2022). Tfcns: A cnn-transformer hybrid network for medical image segmentation. Proceedings of the International Conference on Artificial Neural Networks, Bristol, UK, 6–9 September 2022.

[28] Cao H., Wang Y., Chen J., Jiang D., Zhang X., Tian Q., Wang M. (2022). Swin-unet: Unet-like pure transformer for medical image segmentation. Proceedings of the European Conference on Computer Vision, Tel Aviv, Israel, 23–27 October 2022.

[29] Heidari M., Kazerouni A., Soltany M., Azad R., Aghdam E.K., Cohen-Adad J., Merhof D. Hiformer: Hierarchical multi-scale representations using transformers for medical image segmentation. Proceedings of the IEEE/CVF Winter Conference on Applications of Computer Vision.

[30] Rahman M.M., Marculescu R. Medical image segmentation via cascaded attention decoding. Proceedings of the IEEE/CVF Winter Conference on Applications of Computer Vision.

[31] Zhang Z., Xu C., Li Z., Chen Y., Nie C. (2025). Multi-scale fusion semantic enhancement network for medical image segmentation. Sci. Rep..

[32] Ruan J., Li J., Xiang S. (2024). Vm-unet: Vision mamba unet for medical image segmentation. arXiv.

[33] Huang X., Deng Z., Li D., Yuan X., Fu Y. (2022). Missformer: An effective transformer for 2d medical image segmentation. IEEE Trans. Med. Imaging.

[34] Rahman M.M., Munir M., Marculescu R. Emcad: Efficient multi-scale convolutional attention decoding for medical image segmentation. Proceedings of the IEEE/CVF Conference on Computer Vision and Pattern Recognition.

[35] Wang H., Cao P., Wang J., Zaiane O.R. Uctransnet: Rethinking the skip connections in u-net from a channel-wise perspective with transformer. Proceedings of the AAAI Conference on Artificial Intelligence.

[36] Bougourzi F., Dornaika F., Taleb-Ahmed A., Truong Hoang V. (2024). Rethinking Attention Gated with Hybrid Dual Pyramid Transformer-CNN for Generalized Segmentation in Medical Imaging. Proceedings of the International Conference on Pattern Recognition, Kolkata, India, 1–5 December 2024.

[37] Hu J., Shen L., Sun G. Squeeze-and-excitation networks. Proceedings of the IEEE Conference on Computer Vision and Pattern Recognition.

[38] Woo S., Park J., Lee J.Y., Kweon I.S. Cbam: Convolutional block attention module. Proceedings of the European conference on computer vision (ECCV).

[39] Wang Q., Wu B., Zhu P., Li P., Zuo W., Hu Q. ECA-Net: Efficient channel attention for deep convolutional neural networks. Proceedings of the IEEE/CVF Conference on Computer Vision and Pattern Recognition.

[40] Dai Y., Gieseke F., Oehmcke S., Wu Y., Barnard K. Attentional feature fusion. Proceedings of the IEEE/CVF Winter Conference on Applications of Computer Vision.

